# Insight into Fluorocarbon Adsorption in Metal-Organic Frameworks via Experiments and Molecular Simulations

**DOI:** 10.1038/s41598-019-46269-7

**Published:** 2019-07-16

**Authors:** Dushyant Barpaga, Van T. Nguyen, Bharat K. Medasani, Sayandev Chatterjee, B. Peter McGrail, Radha Kishan Motkuri, Liem X. Dang

**Affiliations:** 1 Energy and Environment Directorate, Pacific Northwest National Laboratory, P.O. Box 999, Richland, WA 99352 USA; 2 Physical and Computational Sciences Directorate, Pacific Northwest National Laboratory, P.O. Box 999, Richland, WA 99352 USA

**Keywords:** Metal-organic frameworks, Computational chemistry

## Abstract

The improvement in adsorption/desorption of hydrofluorocarbons has implications for many heat transformation applications such as cooling, refrigeration, heat pumps, power generation, etc. The lack of chlorine in hydrofluorocarbons minimizes the lasting environmental damage to the ozone, with R134a (1,1,1,2-tetrafluoroethane) being used as the primary industrial alternative to commonly used Freon-12. The efficacy of novel adsorbents used in conjunction with R134a requires a deeper understanding of the host-guest chemical interaction. Metal-organic frameworks (MOFs) represent a newer class of adsorbent materials with significant industrial potential given their high surface area, porosity, stability, and tunability. In this work, we studied two benchmark MOFs, a microporous Ni-MOF-74 and mesoporous Cr-MIL-101. We employed a combined experimental and simulation approach to study the adsorption of R134a to better understand host-guest interactions using equilibrium isotherms, enthalpy of adsorption, Henry’s coefficients, and radial distribution functions. The overall uptake was shown to be exceptionally high for Cr-MIL-101, >140 wt% near saturation while >50 wt% at very low partial pressures. For both MOFs, simulation data suggest that metal sites provide preferable adsorption sites for fluorocarbon based on favorable C-F ··· M^+^ interactions between negatively charged fluorine atoms of R134a and positively charged metal atoms of the MOF framework.

## Introduction

Chlorofluorocarbons were widely used in the past as refrigerants because of their high thermodynamic efficiency, high boiling points, non-toxicity, and low flammability. However, because chlorofluorocarbons contribute to ozone depletion, hydrofluorocarbons (HFC) have emerged as environmentally friendly alternatives. The lack of chlorine in HFCs significantly reduces the ozone depletion potential of the compound. Among them, R134a (1,1,1,2-tetrafluoroethane), a replacement for the commonly used Freon-12 (dichlorodifluoromethane), has been favored in industrial use. R134a is primarily utilized for domestic refrigeration and air conditioning. Its vapor-liquid equilibrium properties and liquid microscopic structure have been well-studied^1^. However, for use with nanoporous sorbent materials, the fundamental host-guest interaction during adsorption of R134a must be thoroughly interpreted to identify more efficient use of such HFCs in industrial applications.

Among the many different classes of nanoporous materials studied for fluorocarbon adsorption, metal-organic frameworks (MOF) are very promising given their high porosity, large surface area, modular structures, and thermal/chemical stability. MOFs have been widely studied for gas adsorption/separation, storage and catalysis applications^2–11^. The building blocks of MOFs consist of inorganic metals that form complexes with organic linkers, both of which can be interchanged to develop a seemingly infinite number of structures. Among the hundreds of known MOF structures, microporous Ni-MOF-74 and mesoporous Cr-MIL-101 have attracted attention because of their high adsorbate affinity, high adsorption capacity, and ease of synthesis compared to other high surface area MOFs. Ni-MOF-74 is an analog of the isoreticular MOF-74 framework originally reported by Rosi *et al*. Containing Ni^+^ ^2^ clusters surrounded by coordinated 2,5-dihydroxyterepthalic acid (DOBDC), this framework contains one-dimensional cylindrical pores (~11 Å) with a high density of open, unsaturated metal sites that have been shown to significantly promote gas adsorption and separation properties^12,13^. In fact, we recently showed excellent R134a adsorption uptakes using Ni-MOF-74 and its pore engineered mesoporous analogs exhibiting high affinity for fluorocarbon at low partial pressures^14,15^. Cr-MIL-101, originally proposed by Ferey *et al*., consists of mesoporous cages (~25–30 Å) connected via microporous pentagonal (~11 Å) and hexagonal (~16 Å) windows^16^. Although the chromium metal centers are fully saturated unlike Ni-MOF-74, the large mesoporous cages of this framework have exhibited excellent light gas adsorption/storage properties^17^. Such well-studied, representative microporous and mesoporous MOFs help to establish new benchmarks and design criteria for novel adsorbents. Therefore, these two MOFs have been considered in this work to interpret the fundamental host-guest chemistry between these frameworks and fluorocarbon R134a adsorbate using experimental methods and computer simulation models.

To date, most simulation studies of MOFs have focused on the adsorption and transport properties of small gases, mainly H_2_, CO_2_, H_2_O and alkanes^3,7,18–28^ and very little attention has focused on fluorocarbons^6,29^. Moreover, only the adsorption of small refrigerants, such as R12, R13, R14, R22, R245fa^6,30^, has been studied experimentally and computationally on these MOFs. The only reported work that has focused on the use of these materials for R134a adsorption are recent publications from our group^15,31^. Therefore, in this work, to understand the global adsorption mechanism and the whole shape of the adsorption isotherm, we carried out gravimetric adsorption experiments and performed grand canonical Monte Carlo (GCMC) simulations to systematically study the adsorption of R134a in microporous Ni-MOF-74 and mesoporous Cr-MIL-101.

## Results and Discussion

**Figure 1:**
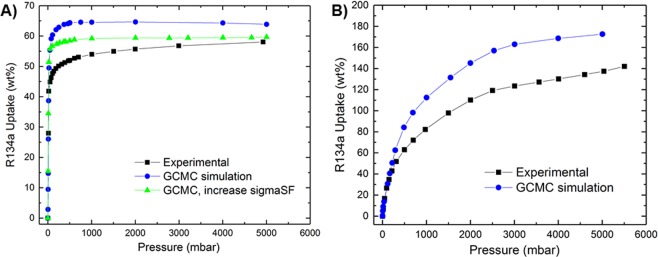
Adsorption of R134a at 298 K in MOFs. (**A**) Ni-MOF-74; (**B**) Cr-MIL-101.

GCMC simulations were performed for R134a adsorption in Ni-MOF-74 at 298 K. We compared the simulated adsorption isotherms with experimentally collected data as shown in Fig. 1A. It can be seen that adsorption isotherms increase with pressure and reach a plateau characteristic of Type-I International Union of Pure and Applied Chemistry (IUPAC) isotherm behavior. As with N_2_ and Ar adsorption (Fig. S1), Ni-MOF-74 is shown to reach saturation with R134a at much lower pressures than Cr-MIL-101. This saturation behavior can be attributed to high affinity unsaturated coordination sites on the metal node which are available in the activated Ni-MOF-74 but not in Cr-MIL-101. The shapes of these isotherms suggest that mechanism of R134a adsorption in Ni-MOF-74 is dominated by the affinity to open metal sites. The simulated isotherm in Fig. 1A overestimates the experimental result by ~15%. The saturated loading measured in our experiment was 57% by weight (g R134a loaded/g adsorbent), while in our simulation it was 65 wt%. This discrepancy between simulation and experimental results has been observed in other simulation works^6,29,32,33^ and can be attributed to the imperfections of the synthesized MOFs as compared to the simulated MOF. Some defects and disorders present in the experimentally synthesized material can result in the decrease of accessible surface area and free volume causing a decrease in adsorption uptake as compared to the perfectly ordered computer-constructed structure of the MOF.

To capture a more reasonable representation of experimental results, the intermolecular interaction potential parameters between R134a and Ni-MOF-74/Cr-MIL-101 used in these simulations could be fine-tuned. As an example, the cross diameter of the Solid-Fluid (sigmaSF) interaction was increased for Ni-MOF-74 by 15%. The resulting isotherm as shown by the green curve in Fig. 1A showed much better agreement with the experimental isotherm. Increasing the sigmaSF results in an increase in the repulsive term and a decrease in the attractive term of the Lennard-Jones potential, which therefore reduces the uptake of R134a in Ni-MOF-74.

Alternatively, the efficacy of the force fields can be improved by using more advanced density functional theory simulations as reference. For example, the reference density functional theory simulations used to develop the force fields for Cr-MIL-101 utilized the BLYP functional^2^. However, recent studies indicate that PBE or PBE0 functionals would be a better choice for organometallic complexes^34^. Therefore, we re-simulated the Cr_3_O trimer of Cr-MIL-101 with the PBE functional using NWChem. The resulting Mulliken charges for Cr_3_O are given in Supplementary Fig. S5^35^. When compared to the corresponding charges obtained with the BLYP functional, the magnitude of charges obtained with the PBE functional are higher for both chromium cations (more positive) and fluorine and oxygen anions (more negative). The increased magnitude of these charges could constrain the degrees of freedom of adsorption of R134a in the host matrix, thereby resulting in effectively increased repulsion between the guest molecule and the host matrix.

Simulated and experimental adsorption isotherms of R134a in mesoporous Cr-MIL-101 at 298 K are presented in Fig. 1B. Similar to Ni-MOF-74 the isotherms exhibit Type-I IUPAC behavior at room temperature. However, unlike Ni-MOF-74, saturation is not obtained even at high pressures. This gradual increase to saturation for Cr-MIL-101, similar to N_2_ and Ar adsorption (Fig. S2), suggests that the mechanism of R134a adsorption over the studied pressure region is largely a function of pore filling. The simulated isotherm agrees very well with experiment at lower pressures (up to 300 mbar), but overestimates the experimental result at higher pressures. The discrepancy between simulation and experimental results increases with pressure. Again, this can be attributed primarily to the difference between our models and the actual sample as well as the potential parameters used in this study. Specifically, this difference at higher pressures for both MOFs can be attributed to the interaction parameters used in the simulation, which were optimized using Henry coefficients derived from low-pressure data. Therefore, it is expected that the calculated high-pressure data, derived from optimized interaction parameters using low-pressure data, would show some discrepancy between experimental and simulated isotherms. Both simulated and experimental data shown in Fig. 1 show that the isotherm of R134a in Ni-MOF-74 exhibits a sharp increase in the uptake at low pressures and then reaches a plateau at about 1 bar, while the uptake of R134a in Cr-MIL-101 increases gradually with pressure and does not saturate, even at 5 bar. This is likely due to saturated adsorption sites and pore filling of smaller micropores and total pore volume in Ni-MOF-74 as compared to larger mesopores/volume of Cr-MIL-101. It is worth noting that at similar high-pressure conditions (6 bar), the experimentally measured uptake capacity of R134a in Cr-MIL-101 is over 140 wt%, which is among the highest uptakes for this fluorocarbon reported to date and more than twice (58%) that observed for Ni-MOF-74. This is because at higher pressures, the free volume is the dominant factor that determines adsorption capacity; Cr-MIL-101 with its larger pore size/volume is capable of storing guest molecules in higher weight percentages than Ni-MOF-74, and a higher pressure is needed to attain saturation in Cr-MIL-101. At very low pressures, where host-guest interactions dominate, the adsorption uptake of R134a in Ni-MOF-74 is significantly higher than Cr-MIL-101. This is indicated by higher uptake of R134a in Ni-MOF-74 in comparison with Cr-MIL-101 at the same pressure as estimated by isotherm fits (Fig. S8). At these lower pressures, (~15 mbar) Ni-MOF-74 adsorbs more than four times the amount adsorbed in Cr-MIL-101 (~33 wt% versus 8 wt%, respectively), probably because of its high density of open metal centers and smaller pore structure that promotes more host-guest interactions.

It was also observed that for both MOFs, the R134a desorption isotherms match closely with the corresponding adsorption isotherms with negligible hysteresis (Fig. S3). This is important for cyclic use of these materials in a closed loop chiller system where the reusability and longevity of the sorbent relies on stable, consistent uptake with each adsorption/desorption cycle. Moreover, the structural integrity of Ni-MOF-74 and Cr-MIL-101 after sorption of R134a was experimentally confirmed by PXRD analysis and comparisons prior to exposure (Fig. S6).

**Figure 2:**
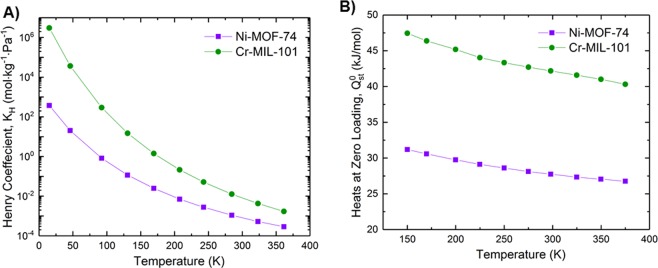
R134a adsorption in MOFs at various temperatures. (**A**) Henry coefficient; (**B**) enthalpy of adsorption at zero loading.

This interaction strength between adsorbate and adsorbent also was characterized using the enthalpy of adsorption at zero loading (${Q}_{st}^{0}$) and an estimation of the Henry coefficient (K_H_). Both were determined from GCMC simulations, and the results are presented in Fig. 2 for various temperatures ranging from 150 K, slightly below the triple point (169.85 K) of R134a, to 375 K, just above the critical point (374.21 K).

It is evident that at 298 K, K_H_ and ${Q}_{st}^{0}$ of R134a in Ni-MOF-74 are 2.8 × 10^−4^ mol/kg/Pa and 27.7 kJ/mol, respectively. For R134a in Cr-MIL-101, K_H_ and ${Q}_{st}^{0}$ are 5.3 × 10^−3^ mol/kg/Pa and 42.2 kJ/mol, respectively. It can be seen from Fig. 2A that the Henry coefficient of R134a adsorption in Cr-MIL-101 is about two orders of magnitude higher than that in Ni-MOF-74 at 150 K, which is below the triple point of R134a (169.85 K). When the temperature increases, the Henry coefficient decreases for both Ni-MOF-74 and Cr-MIL-101, and they approach similar values when the temperature exceeds the critical point (374.2 K). Interestingly, the enthalpy of adsorption at zero loading shown in Fig. 2B shows that Cr-MIL-101 exhibits higher enthalpies than Ni-MOF-74. This indicates a stronger interaction between R134a and Cr-MIL-101 than between R134a and Ni-MOF-74 when the concentration of the fluorocarbon is near zero. As expected, the ${Q}_{st}^{0}$ decreases with increasing temperature, because as the velocities of adsorbate molecules increase, they escape the adsorbent, thereby negatively impacting adsorption.

**Figure 3:**
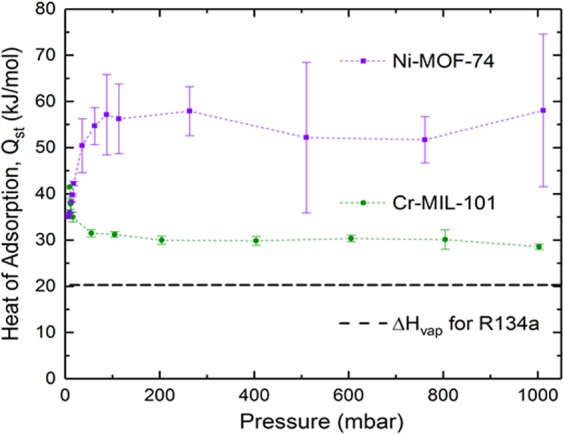
Simulated enthalpy of adsorption of R134a in MOFs at 298 K. The dashed line indicates enthalpy of vaporization of R134a from NIST^45^.

For increasing concentrations of R134a, the adsorption enthalpies in MOFs at 298 K and various pressures were calculated from our simulations and presented in Fig. 3 with error bars shown.

Consistent with Fig. 2, at very low pressures (or low concentration of adsorbate gas) the heat of adsorption for Cr-MIL-101 is higher than that for Ni-MOF-74. The adsorption enthalpy in Ni-MOF-74 quickly increases from ~35 kJ/mol at very low pressures to >50 kJ/mol when the pressure is increased slightly from 10 mbar and then remains almost unchanged at higher pressures due to pore saturation. On the other hand, the enthalpy in Cr-MIL-101 drops quickly from ~42 kJ/mol at very low pressures to ~30 kJ/mol at higher pressure (>100 mbar). This indicates that adsorbate-adsorbent interactions control adsorption at very low loadings and adsorbate-adsorbate interactions control adsorption after the preferential sites have been occupied. Beyond this low pressure region, the adsorption heat in Ni-MOF-74 rapidly increases because of high density of open metal centers and smaller micropores that can hold gas molecules closer to each other, resulting in a more condensed phase. In contrast, Cr-MIL-101 with mesopores that have a much larger capacity do not reached saturation in the pressure range studied; therefore, the adsorbed phase in Cr-MIL-101 is still diluted in comparison with the bulk phase at the same condition, resulting in a lower enthalpy of adsorption than Ni-MOF-74.

**Figure 4:**
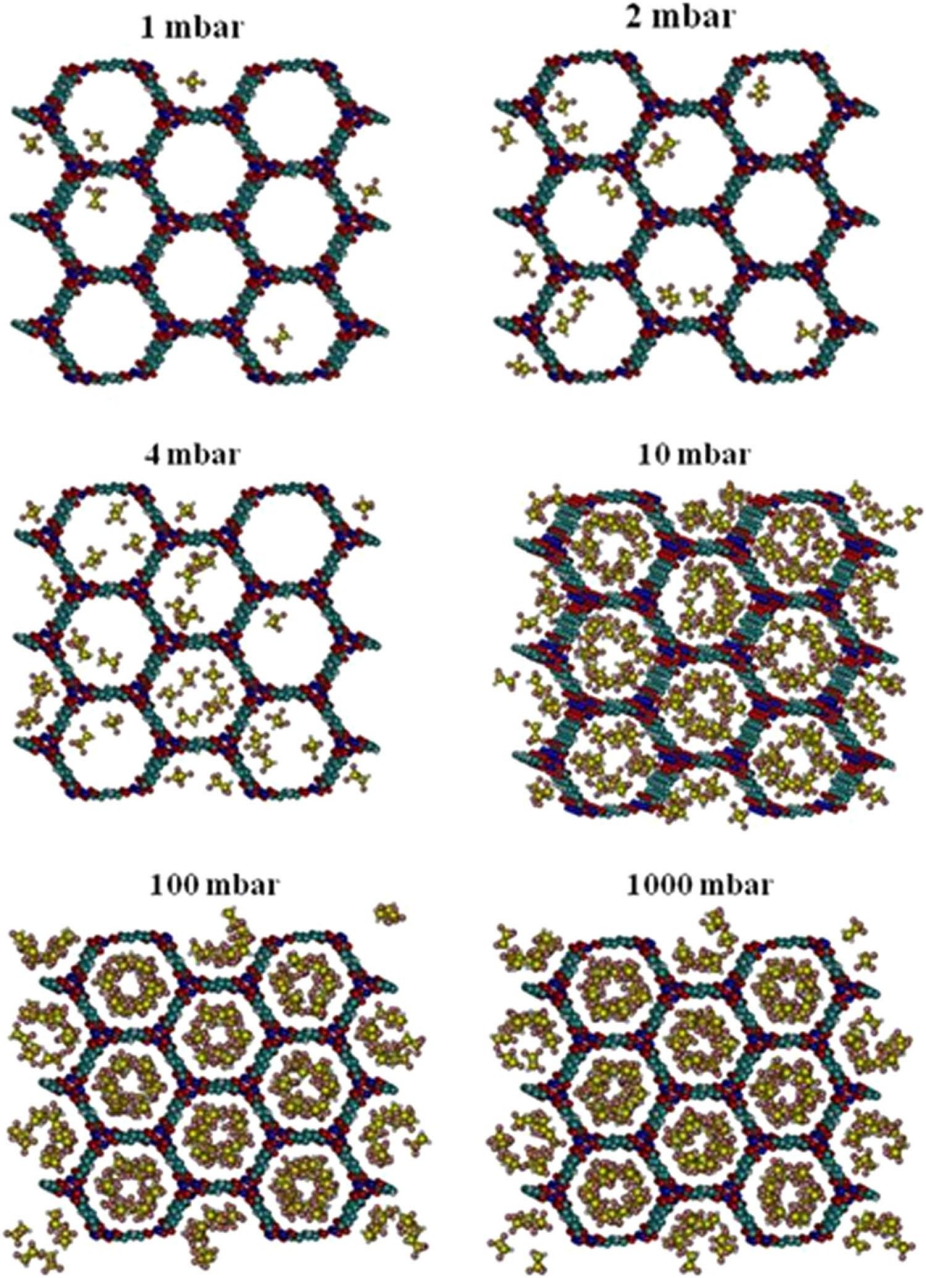
Snapshots of R134a in Ni-MOF-74 at 298 K at various pressures.

To gain a qualitative molecular-level understanding of the behavior of R134a adsorption in each of the two MOFs, we created snapshots of the simulation at various pressures. These snapshots obtained from GCMC simulations of R134a in Ni-MOF-74 at 298 K are presented in Fig. 4. It can be seen that at low pressures such as 1 to 2 mbar, guest molecules are located closer to the nickel sites than the organic linkers due to stronger interactions of negatively charged fluorine atoms with positively charged nickel sites.

As pressure slightly increases, these adsorbed molecules become anchors that attract more molecules to form small clusters due to the hydrogen bonding between fluorine and hydrogen atoms of R134a molecules. As pressure increases, these small clusters of guest molecules start to grow and merge with neighboring molecules to form larger clusters and gradually fill the inner space of the pores.

**Figure 5:**
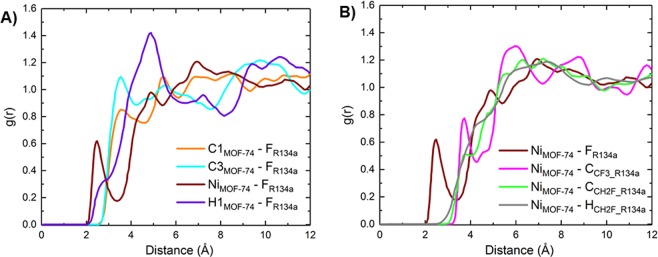
R134a in Ni-MOF-74 at 298 K. (**A**) RDFs between F_R134a and different positively charged atoms of Ni-MOF-74. (**B**) RDFs between nickel and different atoms of R134a.

To gain more insight on the preferential adsorption sites, we computed radial distribution functions (RDFs) between framework atoms and different atoms of R134a. The RDFs of fluorine atoms of R134a molecules around positively charged framework sites and RDFs of different atoms of R134a around nickel sites of Ni-MOF-74 are shown in Fig. 5A,B, respectively. By quantifying the distance between each adsorbate atom and each adsorbent atom, the preferred host-guest interaction can be identified. It can be seen that the furthest left peaks are at r = 2.46 Å between the framework nickel and fluorine atoms of R134a, indicating that nickel atoms are preferential adsorption sites.

**Figure 6:**
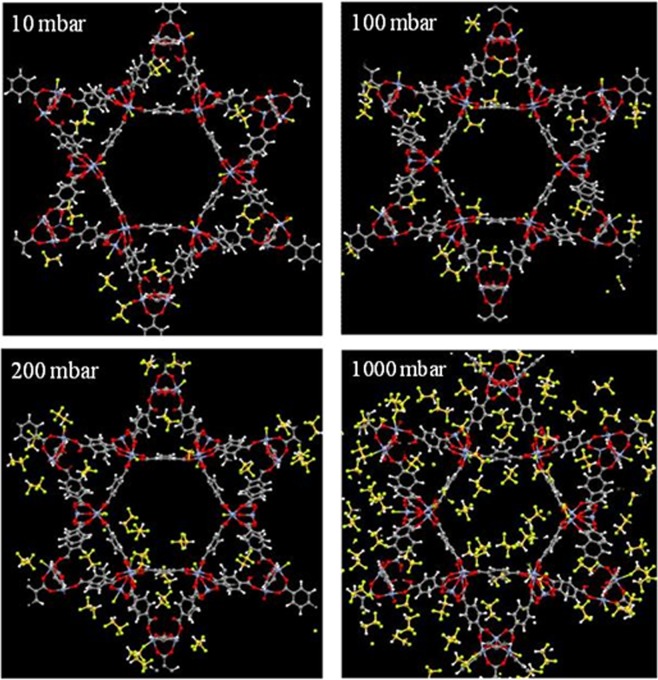
Snapshots of R134a in Cr-MIL-101 at 298 K at various pressures.

Snapshots of R134a adsorption in Cr-MIL-101 at 298 K and various pressures are shown in Fig. 6. At low pressures, guest molecules are mostly adsorbed in the microporous tetrahedron cage due to strong overlap of surface potentials. As pressure increases, R134a molecules begin to appear in the hexagonal window region. When pressure is increased further, the mesoporous cages are gradually filled.

**Figure 7:**
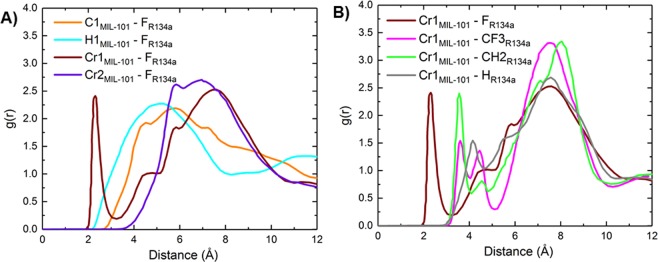
R134a in Cr-MIL-101 at 298 K. (**A**) RDFs between F_R134a and different positively charged atoms of Cr-MIL-101. (**B**) RDFs between unsaturated Cr1 and different atoms of R134a.

The RDFs between different positively charged framework atoms and negatively charged fluorine atoms of R134a are shown in Fig. 7A. The unsaturated Cr1_MIL-101_-F_R134a_ RDF has a very intense first peak at ~2.25 Å, whereas fluorine saturated Cr2_MIL-101_-F_R134a_ peak is broader and shifted further to the right. The intense peak of Cr1_MIL-101_-F_R134a_ clearly indicates a strong favorable binding to the unsaturated chromium atoms of the framework, which are more partially positive compared to the saturated chromium and the linker. This confirms why more R134a molecules are located near the exposed Cr sites than the fluorine saturated Cr sites as seen in the snapshots in Fig. 6. The Fig. 7B presents RDFs between unsaturated Cr1 and different atoms of R134a, which show that fluorine is closer to chromium sites than hydrogen and carbon atoms due to the favorable interactions between F and Cr. It should be noted that consistent with ${Q}_{st}^{0}$ behavior (Fig. 2B), the calculated RDFs show a shorter average bond distance between Cr and F as compared to Ni and F further suggesting that the R134a fluorocarbon has a higher affinity towards Cr-MIL-101 versus Ni-MOF-74 at very low pressures.

## Conclusions

Both experiments and GCMC simulations were conducted to study R134a adsorption in Ni-MOF-74 and Cr-MIL-101 at 298 K. Our simulated isotherms of R134a in MOFs are in good agreement with the experimentally measured isotherms at low pressures but overestimate experiments at higher pressures, which can be attributed to the difference between the solid models and actual materials and the interaction of potential parameters between the host and gas molecules used in this work. Experimentally measured R134a isotherms exhibit Type I IUPAC behavior at room temperature for both MOFs even though saturation is achieved at much lower pressures for Ni-MOF-74.

Simulated isotherms suggested that at the initial stage of adsorption, Cr-MIL-101 exerts a stronger interaction toward R134a than Ni-MOF-74, which is indicated by a higher Henry coefficient and larger enthalpies of adsorption at zero loading. Ni-MOF-74 reaches saturation at a lower pressure than Cr-MIL-101 given the significant differences in pore size and pore volumes. An exceptional saturation capacity of ~140 wt% R134a in Cr-MIL-101 was experimentally observed, which is nearly three times more than that of Ni-MOF-74 (~58 wt%). Beyond initial stages of adsorption, the enthalpy of R134a adsorption in Ni-MOF-74 was shown to asymptotically increase to >50 kJ/mol due to a more condensed phase adsorbed in high open metal centers containing micropores of Ni-MOF-74. In contrast, the adsorbed phase in mesoporous Cr-MIL-101 is less condensed compared with the bulk phase at the same condition, resulting in a lower heats. The snapshots from our simulated and calculated RDFs suggest that the metal sites of both MOFs are more preferable adsorption sites toward R134a than linker atoms because of the favorable C-F … M^+^ interactions between the negatively charged fluorine atoms of R134a and the positively charged metal atoms. Such detailed understanding of host-guest interactions between fluorine atoms and metal centers in MOFs will help in judiciously tailoring new MOF sorbents for various fluorocarbon based adsorption and sensing systems including fluorocarbons with low GWP, zero ODP and longer chain per and polyfluoroalkyl substances (PFAS), perfluorooctaly sulfonates (PFOS)^36^.

## Experimental Methods

### Synthesis procedures

#### Ni-MOF-74

To prepare Ni-MOF-74, 0.218 g (0.75 mmol) of nickel (II) nitrate hexahydrate and 0.704 g (0.375 mmol) of H_2_-dobdc (2, 5-dioxido-1 4-benzenedicarboxylic acid) were mixed (metal: ligand ratio = 2:1) and dissolved in 6 mL of a mixed solvent (DMF: ethanol: H_2_O = 1:1:1 by volume)^37^. The mixture was placed in a Teflon-lined stainless steel autoclave and heated at 373 K for 24 hours. After cooling to room temperature, the product was collected and soaked in methanol for 3 days while replacing fresh methanol every 24 hours.

#### Cr-MIL-101

To prepare Cr-MIL-101, 1 mmol of 1, 4-benzenedicarboxylic acid (H_2_BDC, 166 mg) was added to tetramethylammonium hydroxide solution (5 mL, 0.05 mol·L^−1^) and stirred at room temperature for 10 minutes^38^. To this solution, 1 mmol of chromium (III) nitrate nonahydrate (Cr(NO_3_)_3_·9H_2_O; 400 mg) was added and maintained at pH 6.0–6.5. The reaction mixture was stirred for 20 minutes and then transferred into a 23-mL Teflon-lined autoclave and heated for 24 hours at a temperature of 180 °C. After slowly cooling to room temperature, the green powder that formed was collected by repeated centrifugation and thoroughly washed with distilled water, methanol, and acetone.

### Materials characterization

#### Porosimetry analysis

Nitrogen physisorption was performed on a Quantachrome Autosorb iQ2 unit. Prior to analysis at 77 K, approximately 25 mg of powder sample was outgassed at 423 K under vacuum for 15 hours. BET theory was utilized to derive surface area values, and a single point adsorption at P/P_0_ ≈ 0.99 was used to determine the total pore volume.

#### Crystallinity

X-ray diffraction analyses were performed with a Rigaku MiniFlex 600 X-ray diffractometer. The sample was placed in a powder sample holder under ambient conditions and a pattern was collected from the 2θ range of 3–40°. The step size was 3°/minute. The collected XRD spectra for Ni-MOF-74 and Cr-MIL-101 are shown in Fig. S6.

### Equilibrium R134a adsorption isotherms

The fluorocarbon sorption experiments were performed using an Intelligence Gravimetric Analyzer (IGA) instrument^17^. Prior to measuring the sorption studies, the sample was activated at 473 K under vacuum at a rate of 5 K/minute to remove trapped solvent molecules. The sample was cooled to room temperature, its dry mass was set, and the experimental temperature was maintained by the IGA water bath. The static mode of the IGA was used to measure the sorption studies. The pressure points were set beforehand using the IGA software. The pressure was maintained at the set point by active computer control of the inlet/outlet valves throughout the duration of the experiment. Weight increases resulting from adsorption at each pressure step were plotted against the pressure.

## Simulation Methodology

### Solid models

Both Ni-MOF-74 and Cr-MIL-101 were modeled as rigid frameworks, interacting with gas molecules through van der Waals and Coulombic interactions. The assumption of rigidity is often made when simulating adsorption in MOFs. The crystalline form of Ni-MOF-74 is triagonal and has a space group of $R\overline{3}$ with *a* = *b* = 25.7856 Å, *c* = 6.6883 Å, α = β = 90° and γ = 120°. A unit cell of Ni-MOF-74, whose atomic coordinates were determined from experimental crystallographic data^20^, is shown in Supplementary Fig. S7a. This solid has a uniform 11 Å hexagonal channel with nickel atoms (shown as green spheres in Supplementary Fig. S7a) present at the vertices and organic linkers forming sides of the hexagon. Cr-MIL-101 is built from supertetrahedra (ST) building units that are formed by rigid terephthalic acid linkers and trimeric chromium (III) oxide octahedral clusters^16^. This solid consists of two types of quasi-spherical mesoporous cages (~25–29 Å) connected through microporous pentagonal (~11.7 Å) and hexagonal windows (~16 Å). The crystalline form of Cr-MIL-101 is triclinic and has a space group of *P1* with α = β = γ = 90° and *a* = *b* = *c* = 88.869 Å, which give a very large cell volume of 701,860 Å^3^. In this work, we employed the primitive unit cell of Cr-MIL-101 with α = β = γ = 60° and *a* = *b* = *c* = 62.8399 Å. This primitive unit cell has a volume of 175,465 Å^3^, which is four times smaller than that of the original cubic cell and therefore reduces the computational time while still describes the structure of Cr-MIL-101 equally well (Supplementary Fig. S7b).

### Force fields

Several potential models have been proposed for R134a^39,40^. In this work, we used the model developed by Peguin *et al*.^41^, which used a Lennard-Jones (LJ) 12–6 potential to describe the repulsion and dispersion energy and point charges to describe the Coulombic energy. This model was used to study vapor-liquid equilibrium of R134a using MC simulations by Do *et al*.^1^. The potential parameters of R134a are presented in Supplementary Table S1.

Atomistic representations were used to model Ni-MOF-74 and Cr-MIL-101 in this work (Supplementary Fig. S7), and their corresponding force field parameters are shown in Supplementary Table S2. The LJ parameters for nickel atoms in Ni-MOF-74 were taken from the universal force field^42^, and those for organic ligands were taken from the general Amber force field^30,43^. The force field parameters for Cr-MIL-101 were obtained from the density functional theory studies of Chen *et al*.^2^.

In our simulations, all the LJ cross interaction parameters were determined by the Lorentz-Berthelot mixing rules. LJ potentials were cut off and shifted at a radius of 12 Å. For electrostatic interactions, Ewald summation with a relative precision of 10^−6^ was used for truncation of the Coulombic potentials at a radius of 12 Å.

### GCMC simulations

All simulations in this work were performed using the recently developed RASPA software^44^. We first calculated the void fraction of both structures using the Monte Carlo method by inserting a single helium atom into each framework. The calculated helium void fractions of Ni-MOF-74 and Cr-MIL-101 were 0.74 and 0.809, respectively, which are needed to convert absolute uptake to excess values. We then performed GCMC simulations to study adsorption of R134a in these materials at room temperature. The simulation box consisted of (2 × 2 × 4) unit cells in the case of Ni-MOF-74 and (1 × 1 × 1) primitive unit cell in the case of Cr-MIL-101. Periodic boundary conditions were applied in all three dimensions. The repulsion-dispersion and electrostatic interaction energies between the framework and guest molecule were pre-computed on a three-dimensional grid (with a spacing of 0.2 Å) and stored in a map for later use during the GCMC simulations. For each GCMC simulation run, the chemical potential (µ), volume (V), and temperature (T) were fixed. The chemical potentials of R134a were calculated using the ideal gas law. GCMC simulations were performed with four types of moves: (1) molecular displacement, (2) molecular rotation, (3) insertion of a molecule with random orientation into a random position in the system, and (4) deletion of a randomly chosen molecule from the system. At each pressure point, 10^7^ steps for equilibration were performed, followed by 10^7^ steps to sample thermodynamic property (i.e., the average number of adsorbed molecules per unit cell).

To verify the reliability of our simulation methods, the adsorption of CCl_2_F_2_ (R12) in Ni-MOF-74 at 300 K was performed first as a test and compared to prior experimental and simulated data^6^ as shown in Supplementary Fig. S4. It can be seen that our computed isotherm agrees very well with that obtained by Annapureddy et al. using the MUSIC program^6^, and both simulated isotherms overestimate the experimentally measured isotherm by about 10%. Despite the differences between computer simulations and experimental results, the simulated isotherms exhibit similar trends to those occurring in the experiment. Therefore, we have established that our use of classical force fields can effectively characterize fluorocarbon adsorption in the selected porous materials.

## Supplementary information

Supporting Info
