# Supplementary material for: Insight into Fluorocarbon Adsorption in Metal-Organic Frameworks via Experiments and Molecular Simulations
Source: Sci Rep. 2019 Jul 16;9:10289. doi: 10.1038/s41598-019-46269-7 (PMC6635433; doi:10.1038/s41598-019-46269-7)
Supplement: Supplementary file 1 — Supporting Info [file 41598_2019_46269_MOESM1_ESM.docx]

***Supporting Information***

**Insight into Fluorocarbon Adsorption in Metal-Organic Frameworks via Experiments and Molecular Simulations**

Dushyant Barpaga,^1†^ Van T. Nguyen,^2†^ Bharat K. Medasani,^2^ Sayandev Chatterjee,^1^B. Peter McGrail,^1^ Radha Kishan Motkuri,^1^* Liem X. Dang^2^*

^1^ Energy and Environment Directorate, Pacific Northwest National Laboratory, P.O. Box 999, Richland, WA 99352, USA.

^2^ Physical and Computational Sciences Directorate, Pacific Northwest National Laboratory,
P.O. Box 999, Richland, WA 99352, USA.

^†^Authors contributed equally.

* Corresponding author email: [Radhakishan.Motkuri@pnnl.gov](mailto:Radhakishan.Motkuri@pnnl.gov); [liem.dang@pnnl.gov](mailto:liem.dang@pnnl.gov)

**Porosimetry analysis of synthesized MOFs Ni-MOF-74 and Cr-MIL-101**

**
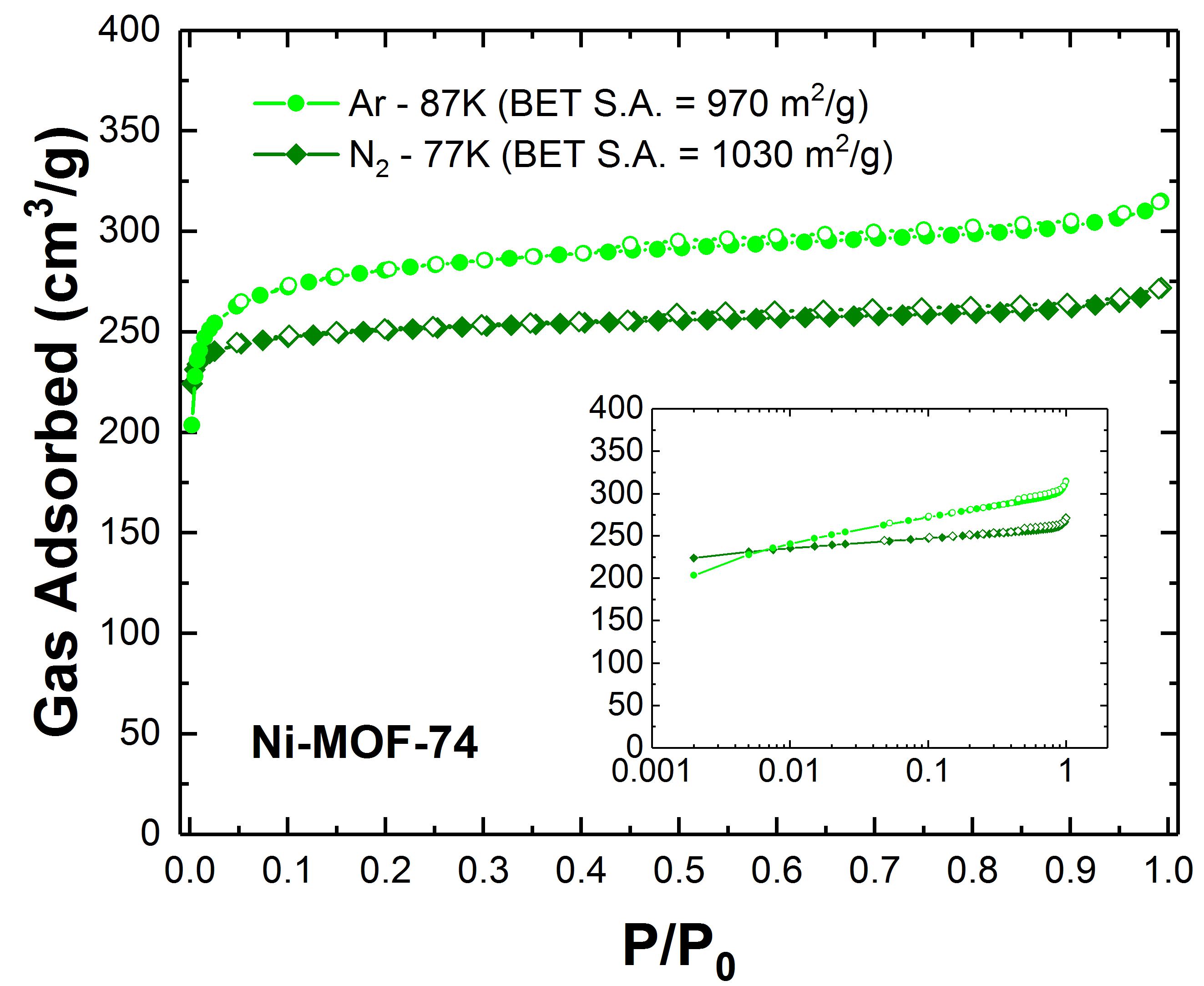
**

**Figure S1.** N_2_ and Ar adsorption (filled) and desorption (unfilled) isotherms for Ni-MOF-74 at 77K and 87K, respectively, with calculated BET surface areas. The inset shows the same plot but in log scale.


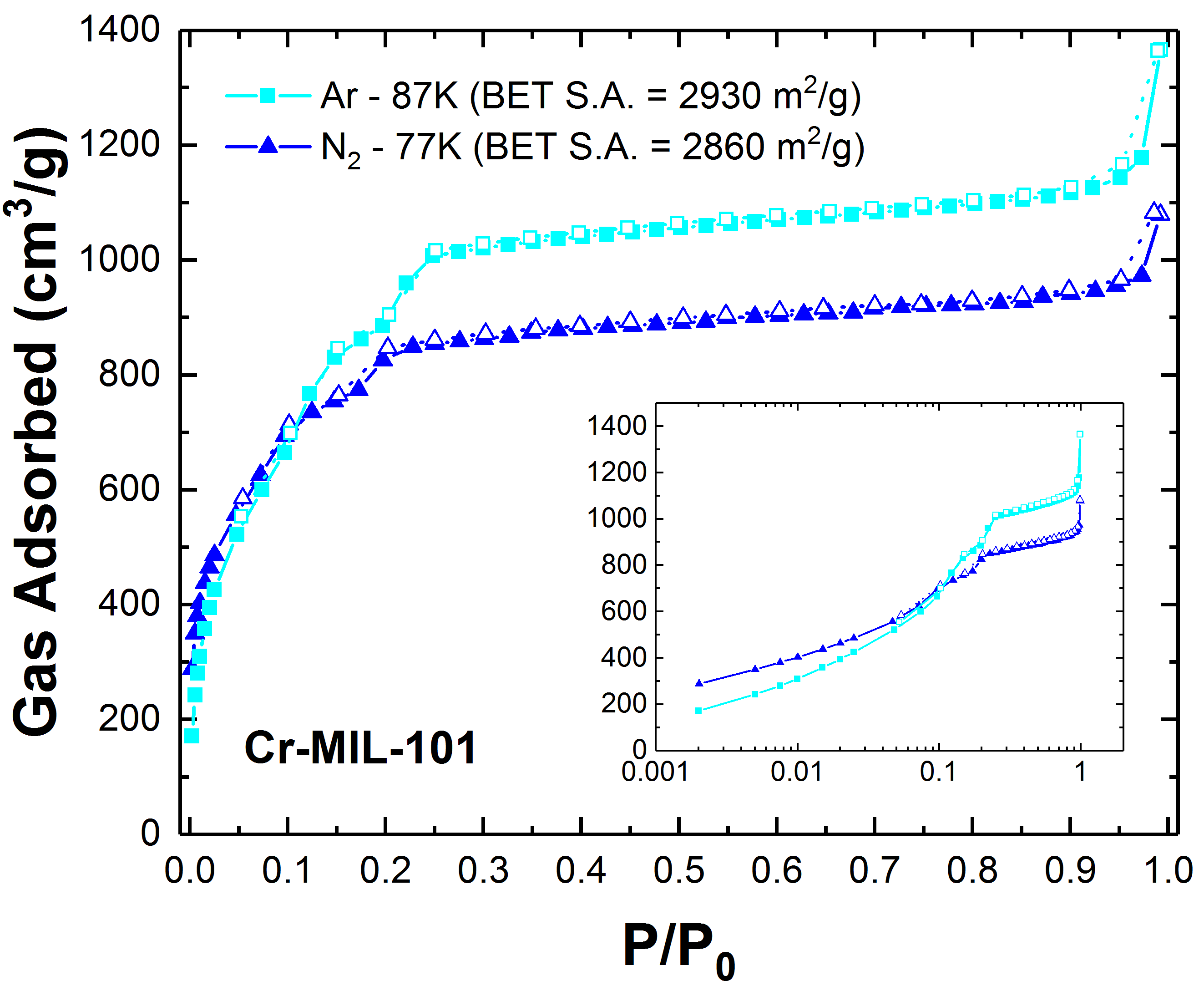


**Figure S2.** N_2_ and Ar adsorption (filled) and desorption (unfilled) isotherms for Cr-MIL-101 at 77K and 87K, respectively, with calculated BET surface areas. The inset shows the same plot but in log scale.

**R134a desorption isotherms showing no hysteresis**


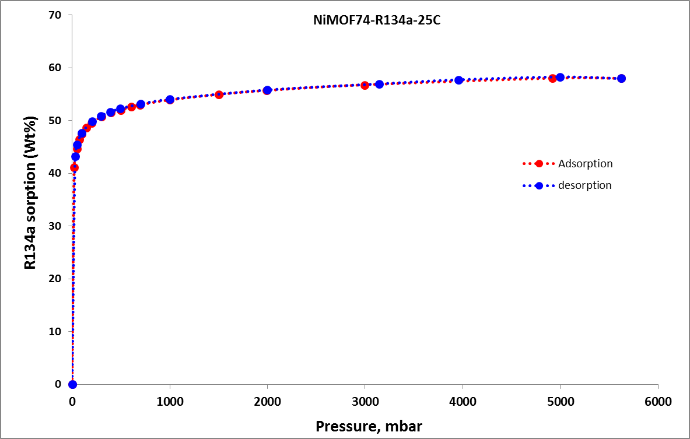


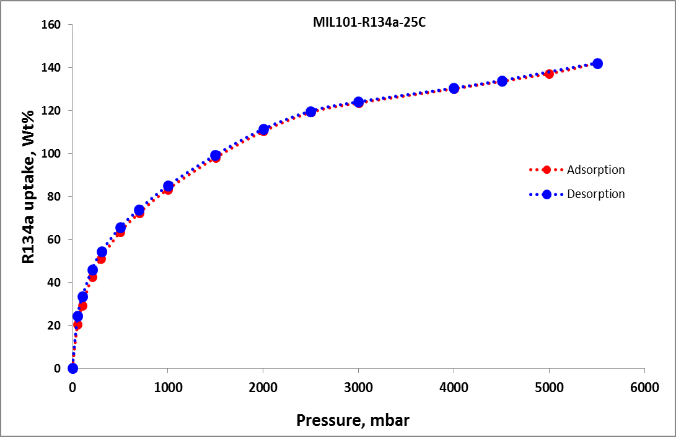


**Figure S3.** R134a adsorption (red) and desorption (blue) isotherms for Ni-MOF-74 and Cr-MIL-101 at 298K, respectively. The isotherms show negligible hysteresis upon desorption.

**Simulation method verification via R12 adsorption**

**
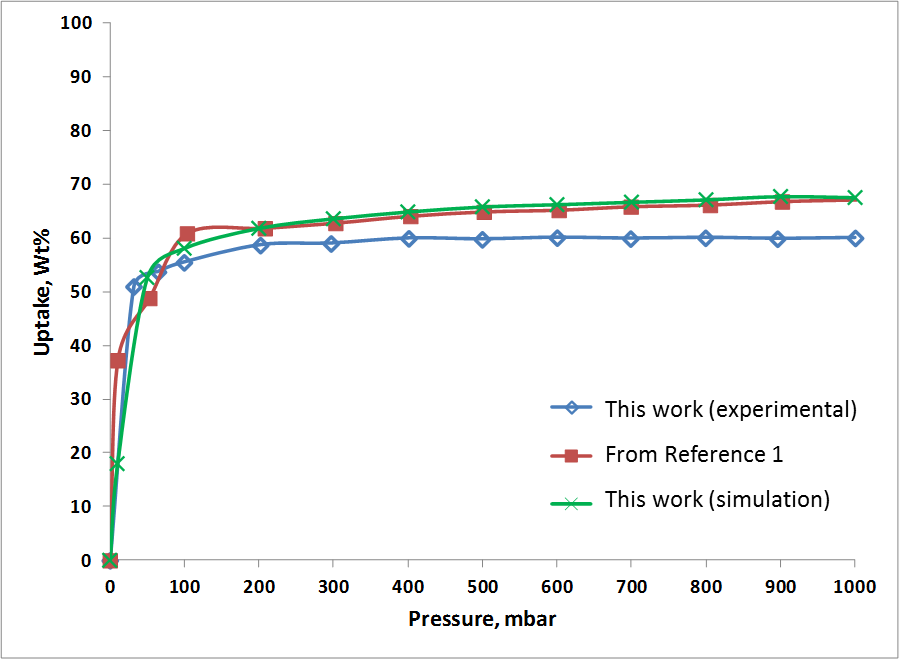
**

**Figure S4.** Adsorption of CCl_2_F_2_ (R12) in Ni-MOF-74 at 300K computed for verification of current simulation technique by comparing with prior results. Reference 1: Nature Communications, 2014, 5, 4368

**Density Functional Theory Simulations for Cr-MIL-101**

**
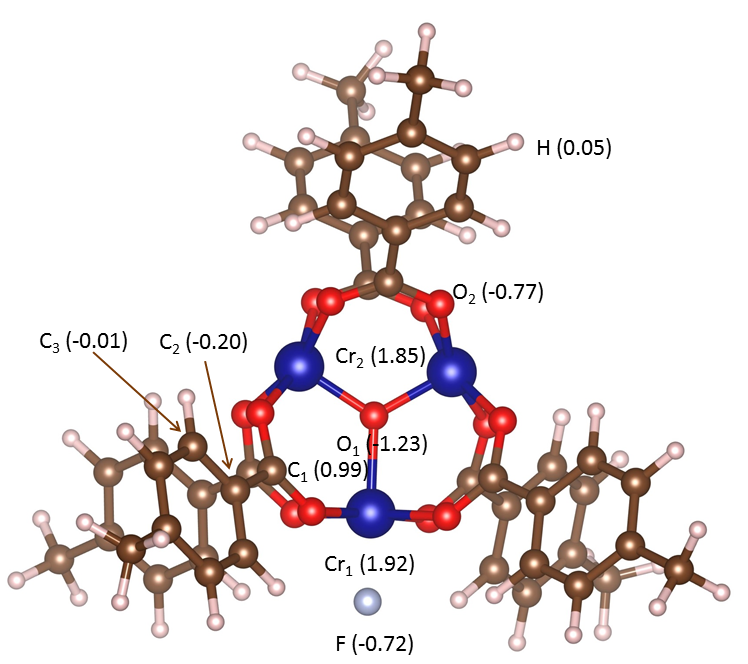
**

**Figure S5.** Mulliken charges of Cr-MIL-101 obtained with density functional theory simulations using PBE functional and cc-pVDZ basis set. The simulations were performed with NWChem software.

**Crystallinity of synthesized MOFs Ni-MOF-74 and Cr-MIL-101**

**
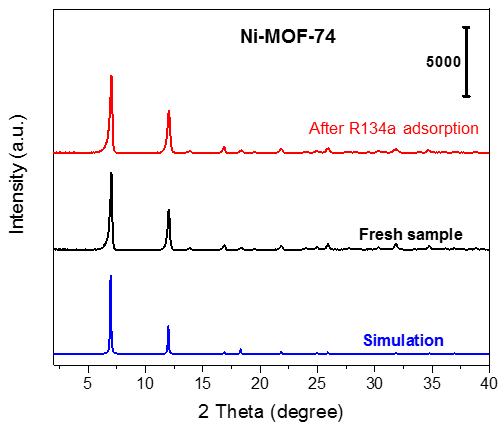

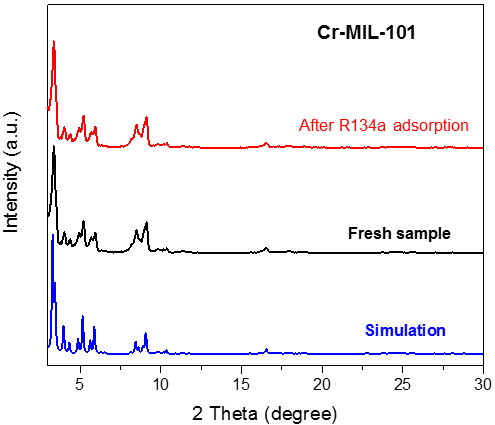
**

**Figure S6.** XRD spectra for Ni-MOF-74 and Cr-MIL-101 before and after R134a adsorption as compared with simulated patterns from published CIF data.

**Unit cells for modeled MOFs**

| 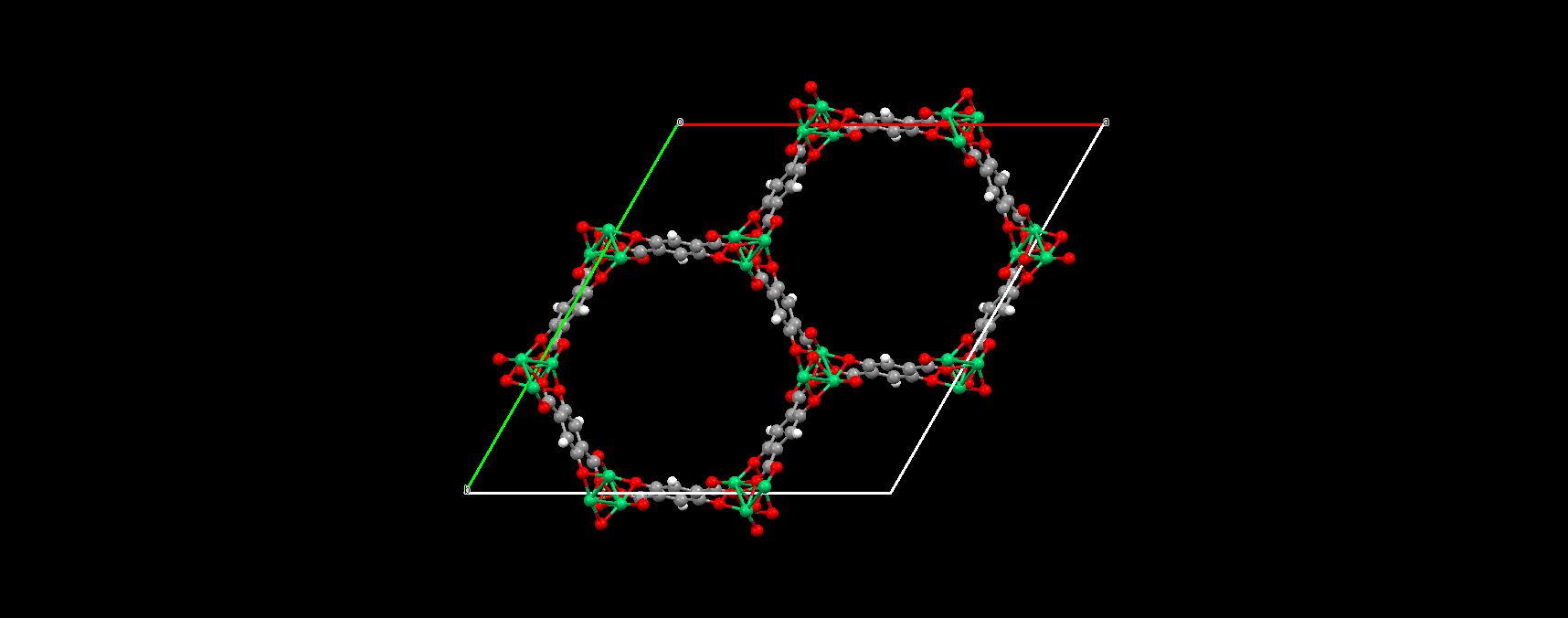 | 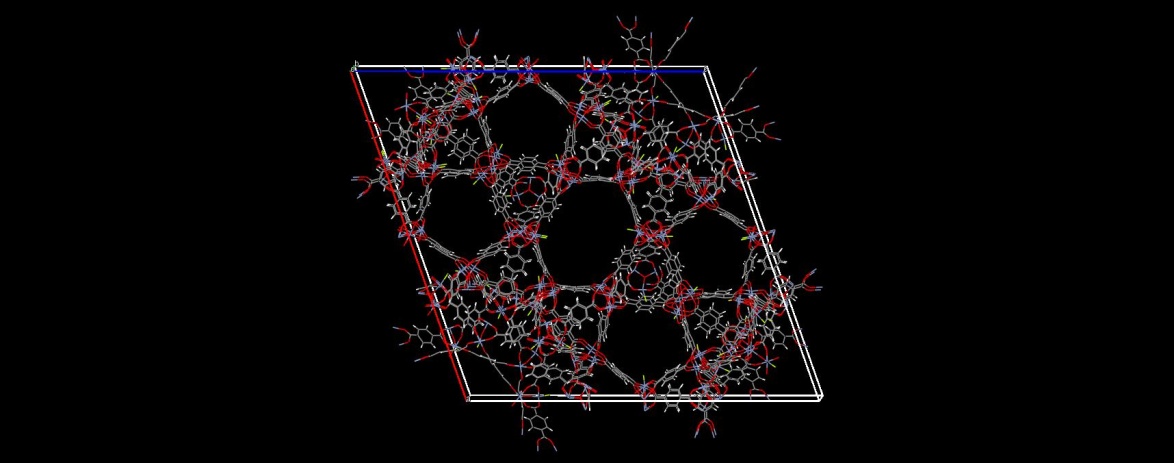 |
| --- | --- |
| (a) | (b) |

**Figure S7.** Unit cells of: a) Ni-MOF-74; b) Cr-MIL-101 primitive. Carbon atoms are represented as silver, H as white, O as red, Ni as green, Cr as purple and F as yellow.

**R134a isotherms at low pressures (zoomed-in from Figure 1)**


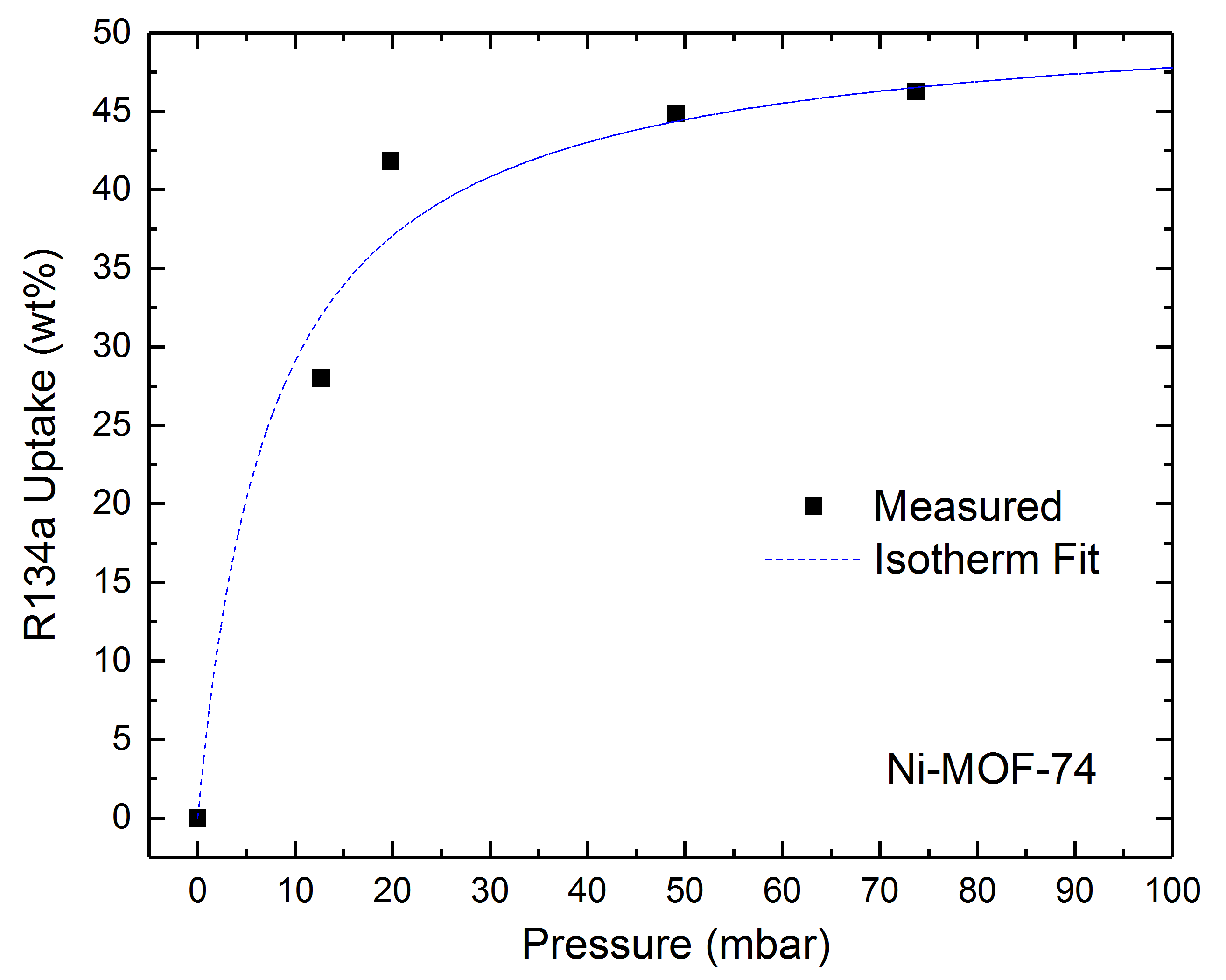

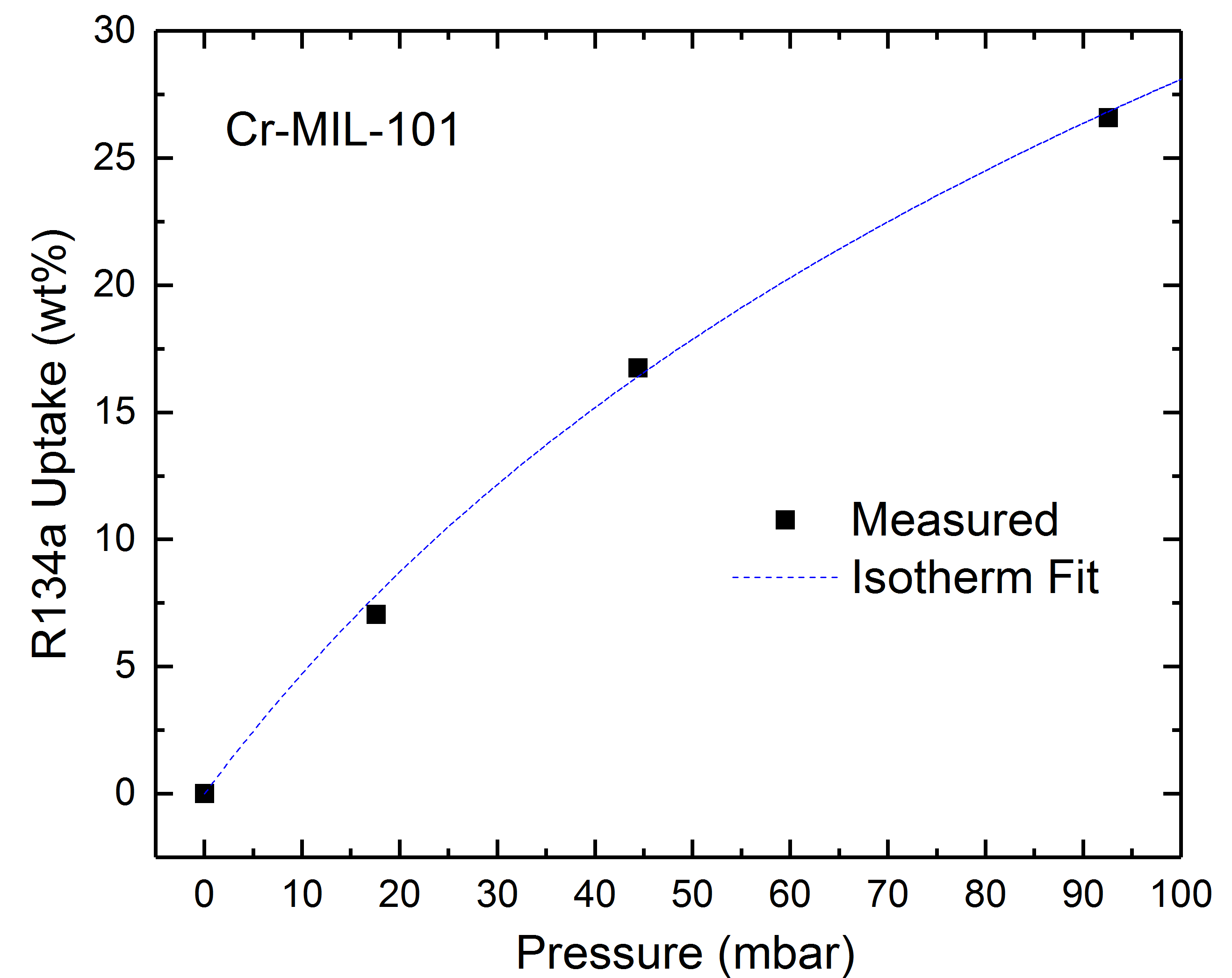


**Figure S8.** Zoomed in plots of R134a isotherms along with corresponding isotherm fits.

**Force field parameters**

**Table S1.** Potential parameters for R134a.

| Atom / Group | *ɛ/k_B_* (K) | *σ* (Å) | *q* (e) |
| --- | --- | --- | --- |
| C (CF_3_) | 47.0 | 3.6 | 0.547 |
| C(CH_2_F) | 47.0 | 3.6 | 0.041 |
| H | 10.0 | 2.5 | 0.104 |
| F | 24.0 | 2.92 | -0.199 |

**Table S2.** Force field parameters for MOFs.

| Ni-MOF-74 | | | | Cr-MIL-101 | | | |
| --- | --- | --- | --- | --- | --- | --- | --- |
| Atom | *ɛ/k_B_* (K) | *σ* (Å) | *q* (e) | Atom | *ɛ/k_B_* (K) | *σ* (Å) | *q* (e) |
| C1 | 43.28 | 3.4 | 0.789 | Cr1 | 7.548 | 2.693 | 1.619 |
| C2 | 43.28 | 3.4 | -0.239 | Cr2 | 7.548 | 2.693 | 1.35 |
| C3 | 43.28 | 3.4 | 0.358 | O1 | 48.158 | 3.033 | -0.853 |
| C4 | 43.28 | 3.4 | -0.201 | O2 | 48.158 | 3.033 | -0.574 |
| H1 | 7.549 | 2.6 | 10.172 | O3 | 48.158 | 3.033 | -0.438 |
| Ni | 7.549 | 2.525 | 1.10 | C1 | 47.856 | 3.473 | 0.496 |
| O1 | 105.685 | 2.96 | -0.65 | C2 | 47.856 | 3.473 | -0.07 |
| OH | 105.685 | 3.066 | -0.679 | C3 | 47.856 | 3.473 | -0.058 |
|  |  |  |  | H1 | 7.649 | 2.846 | 0.108 |
|  |  |  |  | F1 | 36.483 | 3.093 | -0.547 |
